# Home-Based Treatment for Chronic Pain Combining Neuromodulation, Computer-Assisted Training, and Telemonitoring in Patients With Breast Cancer: Protocol for a Rehabilitative Study

**DOI:** 10.2196/49508

**Published:** 2023-11-16

**Authors:** Lorenzo Conti, Chiara Marzorati, Roberto Grasso, Roberta Ferrucci, Alberto Priori, Francesca Mameli, Fabiana Ruggiero, Gabriella Pravettoni

**Affiliations:** 1 Applied Research Division for Cognitive and Psychological Science European Institute of Oncology IRCCS Milan Italy; 2 Department of Oncology and Hemato-Oncology University of Milan Milan Italy; 3 Neurophysiology Unit Foundation IRCCS Ca’ Granda Ospedale Maggiore Policlinico Milan Italy; 4 ASST Santi Paolo e Carlo San Paolo University Hospital Milan Italy; 5 Aldo Ravelli’ Research Center for Neurotechnology and Experimental Brain Therapeutics Department of Health Sciences University of Milan Milan Italy

**Keywords:** telemedicine, chronic pain, breast cancer, tDCS, transcranial direct current stimulation, home care treatment, home care, care, training, pain, cancer, quality of life, rehabilitation, telemonitoring, web platform, exercise, therapy

## Abstract

**Background:**

Chronic pain is a disabling symptom frequently reported in patients with breast cancer with a prevalence ranging from 25% to 60%, representing a major health issue. It has negative consequences on health status, causing psychological distress and affecting quality of life. Furthermore, the clinical management of chronic pain is often inadequate, and many patients do not benefit from the administration of pharmacological treatments. Alternative therapeutic options have been implemented to improve the psychophysical well-being of patients, including neuromodulation and complementary interventions.

**Objective:**

We aimed to investigate the effectiveness of a home care strategy combining computerized rehabilitation, transcranial direct current stimulation (tDCS), and remote telemonitoring via a web-based platform in patients with breast cancer suffering for chronic pain.

**Methods:**

A web-based structured survey aimed at monitoring chronic pain and its effect on psychological functions will be delivered to patients with breast cancer through social media and email. In total, 42 patients with breast cancer affected by chronic pain will be recruited during the medical screening visit. The patients will be randomly divided into 3 treatment groups that will carry out either tDCS only, exercise therapy only, or a combination of both over a 3-week period. All the treatments will be delivered at the patients’ home through the use of a system including a tablet, wearable inertial sensors, and a tDCS programmable medical device. Using web-based questionnaires, the perception of pain (based on the pain self-efficacy questionnaire, visual analogue scale, pain catastrophizing scale, and brief pain inventory) and psychological variables (based on the hospital and anxiety depression scale and 12-item short form survey) will be assessed at the beginning of treatment, 1 week after the start of treatment, at the end of treatment, 1 month after the start of treatment, and 3 months after the start of treatment. The system’s usability (based on the mobile app rating scale and system usability scale) and its involvement in the decision-making process (based on the 9-item shared decision-making questionnaire) will be also evaluated. Finally, at the end of the treatment, a digital focus group will be conducted with the 42 patients to explore their unexpressed needs and preferences concerning treatment.

**Results:**

The study project is scheduled to start in June 2023, and it is expected to be completed by August 2025.

**Conclusions:**

We expect that the combination of tDCS and telemedicine programs will reduce pain perceived by patients with breast cancer and improve their mental well-being more effectively than single interventions. Furthermore, we assume that this home-based approach will also improve patients’ participation in routine clinical care, reducing disparities in accessing health care processes. This integrated home care strategy could be useful for patients with breast cancer who cannot find relief from chronic pain with pharmacological treatments or for those who have limited access to care due to poor mobility or geographical barriers, thus increasing the patients’ empowerment and reducing health care costs.

**International Registered Report Identifier (IRRID):**

PRR1-10.2196/49508

## Introduction

### Background

Chronic pain is related to a multitude of pathological conditions, with a prevalence of 20% to 30% in Europe, and it is considered one of the major health and social problems that our society is facing [1,2]. This disabling symptom is frequently reported in patients with cancer, who often have to deal with pain during their illness. For this reason, the term “chronic cancer-related pain” has recently been used to identify chronic pain caused by the primary cancer itself, metastases, or treatment [3].

In particular, chronic pain has been specifically investigated in patients with breast cancer, with a prevalence that varies from 25% to 60% of patients [4]. Chronic pain has a significant negative impact on the health status of patients with breast cancer that tend to experience higher levels of perceived stress and greater anxiety and depressive symptoms [5,6], thus compromising medical treatment adherence [7].

Moreover, chronic pain has a detrimental impact on daily activities by negatively affecting patients’ quality of life and can cause high degrees of disability as well as the possible onset of psychiatric problems [2]. It can even compromise the ability to work, especially for the younger patients with breast cancer that face a constellation of complex responsibilities, such as family caregiving, professional duties, and financial uncertainty [4,8].

The clinical management of chronic pain is often inadequate since it is still frequently hidden by patients with breast cancer and often neglected by health care professionals [6]. In addition, many patients report several unmet clinical needs and are often referred to multiple specialists without being effectively taken care of by the health system [2,9-12]. Furthermore, it is important to highlight that in many cases, patients suffering from chronic pain do not benefit from the administration of pharmacological treatments. For this reason, alternative therapeutic options have been implemented, including neuromodulation and complementary interventions aimed at improving psychophysical well-being, such as cognitive-behavioral therapy, occupational therapy, physical treatment, and thermotherapy and cryotherapy [13-19]. Indeed, the implementation of complementary interventions can reduce pain intensity and psychological distress by improving the individual’s ability to cope with and manage their pain [13-15,18].

The term “neuromodulation” refers to stimulation techniques that modify the activity of brain circuits [20]. Neuromodulation techniques are now considered an essential tool of precision medicine, and in recent years it has been greatly used in chronic pain management [21-25]. One of the most adopted approaches during clinical trials [26] is transcranial direct current stimulation (tDCS), a noninvasive neuromodulation procedure that uses a low-intensity current capable of modifying the excitability of the cerebral cortex [27].

The restrictions imposed by the COVID-19 pandemic and the mobility limitations that patients face due to chronic pain conditions have brought about awareness of the need to develop new health services based on digital technologies (ie, eHealth). To address these needs, the use of “telemedicine” has been implemented. Telemedicine is a subset of telehealth that uses communications networks for the delivery of health care services and medical education from one geographical location to another, primarily to address challenges like uneven distribution and shortage of infrastructural and human resources [28]. Among other advantages, it has allowed the provision of remote services directly in the homes of patients [29,30].

The development of computerized rehabilitation protocols at home in combination with noninvasive neuromodulation and monitoring by the clinical team could represent an innovative tool for improving the quality of life and life expectancy of patients at a relatively low cost [29]. It has been observed in patients with chronic pain that the improvement of monitoring through a telemedicine-oriented approach capable of guaranteeing continuous and effective communication with health care professionals [31] and an interaction with the patient community can lead to a significant increase in the effectiveness of treatments [32]. eHealth applications appear to be facilitators of the implementation of shared choice processes between doctor and patient, reducing decision-making conflicts and improving patient satisfaction with the treatment process [33].

The growing use of protocols combining neuromodulation techniques and cognitive behavioral interventions through telemedicine, and the possibility of using them in diversified clinical populations, has led to the search for technological ecosystems capable of guaranteeing more personalized treatment approaches [34,35]. Accordingly, a previous project developed an integrated platform, including customized applications and educational and telemonitoring tools for the management of patients with chronic pain [36,37].

### Aims

This protocol is part of the Technopain project aimed at evaluating a new home care strategy combining computerized training rehabilitation, noninvasive neuromodulation, and remote telemonitoring via a web platform. In this study, the use of telemedicine will allow the assessment of psychosocial and physical symptoms related to chronic pain in patients with breast cancer and the identification of its impact on patients’ quality of life and on decision-making related to their own clinical care. In addition, this study aims to investigate the acceptance of the technological approach and the related ethical challenges in order to define a framework of reliability and trust for future therapeutic application.

## Methods

### Procedure

In a first stage, patients will be invited to participate in a mixed method study through social media and email. The delivery and self-administration of questionnaires will take place through a structured survey that can be filled in on a smartphone, tablet, or computer via a web-based platform (Qualtrics). After completing a web-based consent to data processing, participants will receive a link, which will allow them to access a web-based questionnaire aimed at monitoring the repercussions of chronic pain on psychological functions and on the performance of daily life activities. All data will be collected, anonymized, and stored in a password-protected electronic format.

Subsequently, patients who have previously completed the questionnaires will be identified at a screening visit by the doctor and selected according to the eligibility criteria shown in Textbox 1.

**Inclusion and exclusion criteria.**

Inclusion criteria:

- Diagnosed with breast cancer (stage I, II or II)
- Aged ≥18 years
- Undergoing oncological treatments (postconservative or nonconservative surgery)
- Visual analogue scale score >4
- Completed and signed the informed consent form after a detailed explanation of the task and the tools used in the study
- Possess sufficient cognitive skills to support a rehabilitation process mediated by an electronic device
- Montreal cognitive assessment score >15.5 (adjusted for age and years of education) [38]

Exclusion criteria:

- Started new pharmacological treatments or changed current therapies that act on pain within the last month
- Have a bone marrow stimulator or intrathecal drug delivery pump
- Have respiratory, cardiac, metabolic, or other conditions incompatible with at least 30 minutes of light or moderate intensity exercise as assessed by a specialist
- Have severe cognitive impairment
- Have aphasia, dementia, or a psychiatric comorbidity interfering with communication or rehabilitation program compliance
- Have blindness or severe vision problems that may interfere with the use of the tablet
- Possess insufficient knowledge of the Italian language or inability to understand verbal and written instructions
- Concomitant participation in another study or clinical trial involving rehabilitation therapy or administration of an investigational drug

### Questionnaires

Each patient enrolled in the study will be provided with a tablet on loan for use, which will allow the collection of some symptomatologic manifestations and behavioral characteristics relating to the presence of chronic pain. Table 1 describes the questionnaires that will be digitally administered to investigate the perception of pain, the psychological variables, and the patient’s decision-making within the treatment path.

**Table 1:** List of questionnaires administered to each patient with chronic pain.

Questionnaires		Description
**Pain**		
	PSEQ^a^	Measures the perception of self-efficacy in carrying out daily activities, despite the presence of pain [39,40]
	VAS^b^	A 1-dimensional rating scale of pain intensity [41]
	PCS^c^	Investigates catastrophic thinking resulting from the perception of chronic pain [42]
	BPI^d^	Evaluates the severity of the patient’s pain and its impact on daily functioning [43,44]
**Anxiety and depression**		
	HADS^e^	Investigates the presence of anxious and depressive disorders and evaluates their severity [45]
**Health status**		
	SF-12^f^	Measures the patient’s perception of their health status [46]
**Decision-making**		
	SDM-Q-9^g^	Measures the sharing of decisions between doctor and patient [47]
**Usability tools**		
	MARS^h^	Investigates the quality of the app according to engagement, functionality, aesthetics, and information [48]
	SUS^i^	Evaluates the usability of the pain monitoring system [49]

^a^PSEQ: pain self-efficacy questionnaire.

^b^VAS: visual analogue scale.

^c^PCS: pain catastrophizing scale.

^d^BPI: brief pain inventory.

^e^HADS: hospital and anxiety depression scale.

^f^SF-12: 12-item short form survey.

^g^SDM-Q-9: 9-item shared decision-making questionnaire.

^h^MARS: mobile application rating scale.

^i^SUS: system usability scale.

### Randomization

All patients who have signed the informed consent form will be assigned an alphanumeric identification code through which they will be registered in a management software. Randomization is an integral part of the patient registration process and will take place automatically through an integrated randomization block with a 1:1 ratio, and each participant will be randomly assigned by the use of “RAND” formula in Excel (Microsoft Corp) to 1 of the 3 treatment groups described in Table 2.

**Table 2:** Description of the experimental groups.

Treatment group	Description
Group A (tDCS^a^)	Patients will be treated with only tDCS 3 times a week for 3 consecutive weeks.
Group B (exercise therapy)	Patients will be treated exclusively with exercise therapy for 2 or 3 days a week for 3 consecutive weeks, depending on the patient’s characteristics.
Group C (combined therapy)	Patients will be treated by combining tDCS with exercise therapy. They will be subjected to a cycle of tDCS 3 days a week for 3 consecutive weeks. On the days between 1 stimulation and another, the patients will carry out exercise therapy sessions.

^a^tDCS: transcranial direct current stimulation.

### Treatment With tDCS

The delivery of tDCS will be administered through a programmable medical device called “HDCkit” (Newronica). This kit has been specifically designed for both research and clinical use and allows the administration of tDCS for use at home. The HDCkit comprises an “HCDstim” that delivers the tDCS treatment, a touch screen liquid crystal display “HDCprog” that is connected to the HDCStim and controls stimulation settings, and a “Mindcap” that guarantees the correct placement of the electrodes and the reproduction of the appropriate setup when the tDCS treatment is dispensed at home [50]. In the application, the tDCS consists of very low–intensity electric currents that are barely perceptible (1-2 mA) through 2 electrodes (an anode and a cathode) in electroconductive rubber with an area of ​​about 25 cm^2^ integrated inside sponges soaked in physiological solution to facilitate the flow of current.

### Kari System

Patients who plan to undergo exercise therapy will be provided with the kari system (Euleria Health), a CE class 1/m medical device [51], on loan for use. The kari system comprises a tablet with its charger (supplied to all patients), a wearable inertial sensor (IMU) with its magnetic charging cable, a dedicated app that guides the patient through the exercise therapy program, and a kit of 7 elastic bands to facilitate easily positioning the inertial sensor on the body. The IMU is a noninvasive methodology used to collect motion data related to the body section where it is worn. It allows the clinical team to recognize the physical exercise and to verify the accuracy of the movements performed by the patients.

A member of the clinical team will educate each patient individually on the use of the kari system upon delivery of the same so that everyone acquires familiarity and independence in its safe use.

The tablets will also be used for the periodic compilation of questionnaires and rating scales by patients. The data will be saved locally on the device at the end of each training session and then transmitted to the web management system. Once transferred to the web management system, the data are immediately available for consultation by clinical staff.

The results of the clinical and functional evaluations will be collected anonymously on the patient data collection form.

### Intervention

Patients in groups B and C will carry out exercise therapy sessions involving the use of the kari system at home for about 30 minutes a day under the remote supervision of the clinical staff via the web management system. This device allows researchers to remotely configure, customize, and quantitatively monitor an exercise therapy path for patients. A dedicated app guides the rehabilitation patient through the exercise therapy program configured by the professional. Monitoring is guaranteed by the integrated chat and video call functions, as well as by a web application accessible remotely by the therapist in which various quantitative factors related to the therapy can be monitored, such as adherence and program effectiveness.

Patients assigned to groups A and C will receive tDCS treatment, where stimulation parameters will be personalized on the basis of the patients’ clinical characteristics, according to 1 of the methods described in Table 3.

**Table 3:** Description of transcranial direct current stimulation treatment typologies.

Type of stimulation	Description
Spinal stimulation	The active electrode will be placed on the spine in the median position in correspondence with the 10th thoracic vertebra with the reference on the right arm or in correspondence with the somatosensory area [52]. A stimulation intensity of 2.5 mA will be provided for 20 minutes.
Cerebellar stimulation	The active electrode will be placed in correspondence with the cerebellum with the reference on the right arm [53]. A stimulation intensity of 2.0 mA will be provided for 20 minutes.
Brain stimulation	The active electrode will be placed on the left motor cortex with the reference on the right arm or in correspondence with the contralateral supraorbital cortex [54]. A stimulation intensity of 2 mA will be provided for 20 minutes.
Dorsolateral prefrontal stimulation	The active electrode will be placed on the left dorsolateral prefrontal cortex with the reference on the contralateral area [55]. A stimulation intensity of 2 mA will be provided for 20 minutes.

To explore psychological variables and the individual impact of perceived pain, patients will be evaluated and receive a link with the questionnaires described in Table 1 at the following specific time points: before starting any treatments, 1 week after the start of the treatment, at the end of the 3 weeks of treatment, 1 month after the start of the treatment, and 3 months after the start of treatment. Furthermore, at the end of the treatment, each patient will be asked to answer questionnaires aimed at evaluating the usability of the system.

Finally, a focus group will be conducted with all the patients who took part in the study. The patients will be divided into groups of 14 participants based on the treatment group to which they were assigned. The aim of this investigation is to analyze the unexpressed needs, expectations, and preferences related to the home-based treatment. The focus groups will be conducted starting from a list of questions concerning the experience of the disease by the study participants, the relationship between the painful symptoms and the psychological significance, the expectations with respect to the treatment, and any difficulties encountered during the use of the technologies adopted during the rehabilitation program.

### Focus Group Meetings

A telematic platform will be used to carry out the focus group meetings at the end of the treatment. The duration of the focus group meetings will be between 60 and 90 minutes, and breaks will be provided every 45 minutes to avoid tiring the patients. Audio and video will be recorded from every meeting and subsequently analyzed using T-Lab software, which comprises several linguistic and statistical tools [56].

### Sample Size

At least 42 patients with breast cancer will be recruited for the study. The patients will be divided into 3 experimental groups, each consisting of 14 patients, based on the type of treatment performed (tDCS only, exercise therapy only, or combined treatment). In case of dropouts, the recruitment of patients will continue until 42 participants complete the treatment.

Since the present study is the first to evaluate the effects of tDCS in patients with different types of chronic pain over a 3-week period, to estimate the expected difference between the effect of tDCS and combined therapy (tDCS and exercise therapy), for the calculation of the sample size we relied on a recently published study [54] of 45 patients with fibromyalgia to calculate the sample size. The published study compared the effects of tDCS and exercise therapy if carried out individually or in combination for 1 week. The improvements observed in the 3 treatment groups are shown in the Table 4.

**Table 4:** Mean visual analogue scale (VAS) scores of the different treatment groups.

Treatment group	Baseline VAS	Improvement (%)	VAS at 1 week after treatment	ΔVAS (beginning of treatment vs 1 week after treatment)
tDCS^a^	7.2	18	5.904	1.296
Combined therapy	7.3	40	4.4019	2.8981
Exercise therapy	6.8	24	5.202	1.598

^a^tDCS: transcranial direct current stimulation.

The sample number for each individual clinical center was calculated to demonstrate the difference between the change in the visual analogue scale (VAS) value at the beginning of treatment and after 1 week of treatment between the group of patients receiving tDCS and that receiving combined therapy. A Wilcoxon-Mann-Whitney test was performed using G*Power V3 software [57] with an effect size of 1 and assuming the following parameters: a VAS change of 1.3 in the tDCS treatment group and 2.9 in the combined therapy group, as reported by Mendonca et al [54] in the evaluation after 1 week of treatment; an SD of 1.27 in both groups; a type 1 error of 0.05; and a power of 80%. A sample of the same size is adequate to demonstrate the difference in VAS between the exercise therapy group and the combined therapy group (based on data from Mendonca et al [54]).

### Statistical Analysis

To evaluate the efficacy of the treatments, a statistical analysis will be conducted on the clinical scales, functional tests, and questionnaires using a dedicated software. Data distribution will be evaluated using Shapiro-Wilk tests. In the case of a normal distribution, statistical significance will be evaluated by applying parametric *t* tests and ANOVA for repeated measures. In case the assumption of a normal distribution is not supported, a nonparametric Wilcoxon-Mann-Whitney test will be used. Statistical analysis will be performed on all participants who complete the treatment required by the protocol. Participants who do not complete all phases of the study (ie, dropouts) will be excluded from the data analysis. Furthermore, as suggested by recent scientific literature, Bayesian statistics will also be applied using JASP open-source software in order to better estimate the possible absence of an effect of therapeutic strategy [58]. In particular, intragroup comparison analysis will be assessed using repeated measures ANOVAs (within subjects, time factors). Comparison of the subgroups (n=14 per group) will be performed using parametric or nonparametric hypothesis tests depending on the normality of the data distribution and corrections of type 1 errors. Finally, correlations will be performed between the VAS, patient demographics, and clinical data. All secondary outcomes will be evaluated by descriptive statistical analysis. Analysis will be carried out using SPSS Statistics (version 27; IBM).

### Ethical Considerations

This protocol was approved by the ethical committee of the Istituti di Ricovero e Cura a Carattere Scientifico European Institute of Oncology (April 2023). Written and verbal information will be provided and informed consent will be obtained from all participants. Participation in the study is voluntary and free. No additional fees will be charged to the participants, and no payments will be made.

All the methodologies mentioned in the study protocol will be carried out according to the relevant guidelines and regulations.

### Data Protection

Patient data will be treated as strictly confidential and subject to anonymity so that it cannot be associated in any way with the individual. For this purpose, at the beginning of the study, the study doctor will assign to the participants a code with which they will be identified during all subsequent phases of the study. The data relevant to the study, with the exception of patients’ names, will be recorded, processed, and stored together with this code and all clinical data relating to the state of health. Only the physician and authorized parties will be able to link this code to the patients’ names.

This information will be treated as strictly confidential in accordance with EU Regulation 679/2016, Legislative Decree 196/2003 as amended (Processing of Personal Data) and will only be used for research purposes in connection with this study.

## Results

The project, Technopain, is scheduled to start in June 2023, and it is expected to be completed by August 2025. Results will be available following data analysis in the subsequent months.

This innovative approach will contribute to furthering our understanding of the neurophysiological mechanisms of chronic pain and the related psychosocial and physical symptoms. Additionally, the suitability of the telemedicine approach for patients and clinicians will favor the application of new technologies able to improve patients’ well-being and access to care.

## Discussion

### Projected Impact

The present study protocol represents an innovative approach to the management of chronic pain in patients with breast cancer without the adoption of pharmacological treatments. Consistent with previous studies [15,59-62], we expect that the implementation of combined therapeutic interventions can directly produce a relevant improvement on pain relief.

Considering the beneficial effects of tDCS in increasing emotional well-being in psychiatric and neurological patients [63-65], we also assume that tDCS combined with telerehabilitation programs will reduce psychological distress and improve mental wellness in patients with breast cancer with chronic pain. Moreover, this interactive approach may motivate patients to increase treatment compliance, thus favoring better clinical outcomes.

Another key objective of this integrated home care strategy will focus on limiting the impact of physical and psychosocial symptoms associated with chronic pain on patients’ daily lives [4,7]. They represent significant barriers that can affect the ease of travel to health facilities and cause difficulties in accessing care treatments, with consequent economic and organizational impacts [66]. We hypothesize that the introduction of a home-based approach directly and continuously monitoring patients at home will allow us to assess disease progression and improve patients’ participation in routine clinical care, thus reducing disparities in accessing health care. Moreover, we expect to significantly optimize the rehabilitation session in terms of time and efficacy, thus reducing the direct and indirect health care and social costs.

Finally, the project aims to support patients affected by chronic pain through decision aids during critical moments of the therapeutic journey [67]. Telemonitoring of remote patients throughout the care process allows the evaluation of both clinical outcomes and patients’ reported opinions. Furthermore, the focus groups will allow a greater exchange of the preferences and perceptions of the treatment received by patients with breast cancer, highlighting the strengths and barriers that could have influenced their psychoemotional well-being and their overall experience with chronic pain [68]. We expect that focus group sessions will also contribute to examine adherence, identifying patients who need more personalized pain management strategies, and contextualizing the problem of unrelieved pain.

### Clinical Implications

It is possible to hypothesize several advantages following the application of this treatment. First, we expect a reduction in pain perceived by patients, since noninvasive brain stimulation methods and exercise therapy have been shown to have beneficial effects on the management and treatment of chronic pain in previous studies [15,17,18]. Furthermore, the information obtained may also be useful in the future for other people suffering from chronic pain, allowing us to determine which treatment among those investigated in the study will be more appropriate.

Additionally, we expect that the implementation of a home-based care system can facilitate patient adherence by reducing travel costs and establish a more direct and continuous exchange of information between patients and health care professionals.

### Study Limitations

A main limitation of the present study could be the personal use of the home care strategy. Despite the many advantages of this technological approach, some patients may find it difficult to use the equipment without direct assistance from a health care professional. The self-management of the home care strategy could also have a negative influence on treatment adherence, causing possible dropouts during the study phases. For these reasons, it will be necessary to monitor patients’ motivation during treatment to prevent patients from neglecting rehabilitation.

Patients should also be adequately informed of the possible risks linked to the administration of the treatments to reduce concerns about the occurrence of possible adverse effects of tDCS. In particular, adverse events could involve the appearance of local effects of the stimulation consisting of a slight burning sensation or skin redness. Nevertheless, it should be noted that participation in a trial involves strict control by the experimenter to limit any problems that may arise.

Furthermore, the initial recruitment via social media and email could orient the sample toward younger and technologically experienced patients. However, we adopted this instrument to promote the recruitment of patients throughout the country to overcome geographical barriers related to the access to health care. Additionally, due to the use of digital devices during the rehabilitation program proposed in this study protocol, the enrollment of technologically savvy patients with breast cancer could facilitate the use of the home-based treatment.

Finally, given the importance of subjective perception on the classification of pain, we chose to adopt several self-report tools to assess the personal experience of the patients with breast cancer. However, these instruments have limitations, as it is not possible to obtain objective information. When drawing conclusions, it will therefore be necessary to consider that the data may be influenced by various personal factors.

### Conclusion

The Technopain protocol is a new home care strategy that combines computerized training rehabilitation, noninvasive neuromodulation, and remote telemonitoring via a web platform for the treatment of psychosocial and physical symptoms related to chronic pain in patients with breast cancer. In particular, this approach will evaluate the impact of chronic pain on patients’ quality of life and will offer a rehabilitation program that can favor the treatment of the greatest number of patients through home delivery. This trial will primarily evaluate the fluctuation of perceived pain by patients during and after treatments, evaluating which of the different types of treatments is the most effective between the single treatments or their combination. In particular, we expect that this study will provide a useful treatment for all patients who cannot find relief from chronic pain with pharmacological treatments. Our ultimate aim is to encourage the development of personalized telemedicine, which can provide daily clinical assistance to patients, eliminating the economic and geographical barriers related to the use of health services.

## Abbreviations

tDCS: transcranial direct current stimulation
VAS: visual analogue scale

## Data Availability

Data sharing is not applicable to this protocol as no data sets have yet been collected or analyzed.
